# Groups and Individuals: Optical Flow Patterns of Broiler Chicken Flocks Are Correlated with the Behavior of Individual Birds

**DOI:** 10.3390/ani11020568

**Published:** 2021-02-22

**Authors:** Sabine G. Gebhardt-Henrich, Ariane Stratmann, Marian Stamp Dawkins

**Affiliations:** 1 Center for Proper Housing of Poultry and Rabbits, Division of Animal Welfare, University of Bern, Burgerweg 22, 3052 Zollikofen, Switzerland; sabine.gebhardt@vetsuisse.unibe.ch (S.G.G.-H.); ariane.stratmann@vetsuisse.unibe.ch (A.S.); 2 Department of Zoology, University of Oxford, John Krebs Field Station, Wytham, Oxford OX2 8QJ, UK

**Keywords:** broiler chickens, group welfare, animal welfare, flock behavior, optical flow, precision livestock farming, latency-to-lie

## Abstract

**Simple Summary:**

Technology on farms potentially brings the benefits of improved animal welfare and productivity as well as reduction in disease, waste and environmental impact. However, it also raises public concern about the welfare of individual animals, particularly when applied to large groups such as broiler (meat) chickens. We here address this issue by showing that camera technology can both provide life-long continuous monitoring of the welfare of whole flocks and also give crucial information about the individuals making up the flock. The cameras detect variation between individuals and are sensitive to birds moving abnormally. By testing samples of birds individually, we show that on average slow-moving birds came from flocks that moved slowly overall and showed large variation between individuals whereas on average fast-moving birds came from more active flocks that moved more uniformly. Properly used, camera technology can thus monitor the welfare of flocks continuously throughout their lives and is correlated with the behavior of individual birds.

**Abstract:**

Group level measures of welfare flocks have been criticized on the grounds that they give only average measures and overlook the welfare of individual animals. However, we here show that the group-level optical flow patterns made by broiler flocks can be used to deliver information not just about the flock averages but also about the proportion of individuals in different movement categories. Mean optical flow provides information about the average movement of the whole flock while the variance, skew and kurtosis quantify the variation between individuals. We correlated flock optical flow patterns with the behavior and welfare of a sample of 16 birds per flock in two runway tests and a water (latency-to-lie) test. In the runway tests, there was a positive correlation between the average time taken to complete the runway and the skew and kurtosis of optical flow on day 28 of flock life (on average slow individuals came from flocks with a high skew and kurtosis). In the water test, there was a positive correlation between the average length of time the birds remained standing and the mean and variance of flock optical flow (on average, the most mobile individuals came from flocks with the highest mean). Patterns at the flock level thus contain valuable information about the activity of different proportions of the individuals within a flock.

## 1. Introduction

The use of automated methods for assessing animal welfare is a rapidly growing feature of livestock agriculture [1,2,3,4,5], but commercial poultry farming has raised particular problems because of the large numbers of animals involved. The practical problems of identifying, tracking and locating the many thousands of animals found in large commercial poultry flocks has led to the development of automated systems that do not identify animals as individuals and instead give welfare outcomes that apply to whole groups. For example, flock-level analyses of visual images [6,7,8,9,10] and flock sounds [11,12] deliver useful information on the state of the flock as a whole, but not on individual animals. However, such group level approaches to welfare assessment have been challenged on the grounds that they overlook the most crucial element of all—the welfare of individual animals [13,14]. The aim of this paper is to show that, properly used, automated group-level measures of welfare can contribute to the assessment of individual bird welfare, even without specifically identifying individuals.

Automated systems derive their usefulness from their capacity to collect much more detailed and more continuous information than is possible for a human observer. They enable a stockperson to have 24/7 information about their animals and to have their attention immediately drawn to a problem flock that needs more detailed human inspection or intervention. In a general sense, then, their use as an extension to the work of a good stockperson has the potential to lead to an increase in the welfare of individual animals even where the automated system itself does not distinguish between individuals. 

More specifically, group-level automated systems can also provide more than a simple record of the average behavior of an entire group. Optical flow patterns made by broiler chicken flocks, for example, contain information not only about the average or mean amount of movement within a flock but also how much variation there is in that movement [6,15]. Such variations are an important part of assessing the state of individual animals within a flock and can be described in a range of ways in addition to a simple measure of variance. The skew of a distribution, for example, is a measure of whether the mode is above or below the mean [16]. The skew statistic from optical flow patterns of broiler flock movements can therefore be used to indicate whether the majority of birds in a flock are more or less active than average. An even more informative way of describing variation is the kurtosis, which is a measure of whether there are abnormally long “tails” or outliers to a distribution. The kurtosis of optical flow in broiler chicken flocks indicates whether there is a “tail” of an abnormally large number of very fast (or very slow) movement events and so can be an indication of whether the most active (or most inactive) birds are in the majority or form a tiny minority. In fast-growing broiler chicken flocks, kurtosis reflects the activity of the most active individuals in the flock [15] and is correlated with key welfare outcomes including gait, mortality, pododermatitis and hockburn [17,18,19,20]. While not describing the state of each individual in a flock, optical flow can thus indicate the proportion of birds to which the overall flock measures apply. 

We tested the hypothesis that the group level outputs (mean, variance, skew and kurtosis of optical flow) correlate with the average behavior of a sample of individual birds as assessed in tests designed to measure their movement as individuals. Two of the tests involved the time taken by an individual bird to move down a runway, either with or without obstacles. The length of time a chicken takes to move down a runway towards conspecifics has been used both as a measure of social attraction and also of physical ability to move, particularly where chickens have to overcome obstacles [21,22,23]. We use it here as a measure of activity, however it is caused. The third test was the time taken by a bird to sit down in a shallow water bath (the latency-to-lie test), previously shown to be associated with lameness and poor gait scores [24,25]. Specifically, we predicted that the birds that moved most quickly down the runways and remained standing for the longest time in the water test would come from flocks with the highest mean and the lowest skew and kurtosis. 

## 2. Materials and Methods

### 2.1. Ethical Considerations

All animals were being raised as commercial agricultural livestock in Switzerland under the specifications of the welfare label BTS, which dominates the Swiss market (over 90% of Swiss broilers are produced under this label). The houses all had enclosed outside areas called winter gardens that the chickens could access through popholes. No popholes were opened until the birds were at least 22 days old, after which it was a BTS requirement that they must be opened if the outside temperature was at least 13 °C (days 22–29) and 8 °C (from day 30 onwards). In addition to the winter garden, the BTS also specifies that there must be a minimum of 15 lux of daylight (which can be supplemented with artificial light) and, from the 10th day onward, for the birds to have access to elevated platforms, which increased the available surface by 10%. Cameras were installed in the houses when the houses were empty to avoid disturbance to the birds. The work was approved by the Canton of Bern (BE97/16, on 30 September 2016) and met all cantonal and federal regulations for the treatment of animals.

### 2.2. Animals and Farms

We selected 3 out of 5 farms that were used in a previous study [19] and that had a history of both *Campylobacter* positive and negative flocks according to tests at the abattoir. The 3 farms belonged to one company (Bell AG, Zell, Switzerland). Chicks (Ross 308) were placed “as hatched” (mixed sex) as day-olds and were grown to a maximum stocking density of 30 kg/m^2^ when including the surface of the raised platforms. 

In total, 20 flocks were tested, although complete optical flow records were obtained for only 18. Flock sizes ranged from 11,934 to 24,000 birds (mean: 18,533.7, STD: 3923.0, *n* = 20) and birds were grown to an age of 30 (2 flocks), or 36 (14 flocks with and without thinning), or 37 (4 flocks with thinning) days.

### 2.3. Behaviour Tests for Individual Welfare Assessment

All behavior tests were carried out “blind”, that is, before the optical flow results or the results from the abattoir were known. On the day of the test, birds were between 23 and 28 days of age (mean: 25.5, STD: 1.32). First, a conveniently chosen group of about 20 birds was separated from the flock by a catching frame (114 × 114 × 60 cm^3^) inside the barn with visual contact to the flock. Noticeably lame or sick birds were excluded. Sixteen randomly chosen birds from this catching frame were marked with color on the head, wings, or tail and one after the other underwent the runway tests. After the tests, chicks were weighed, sexed, and scored for pododermatitis and hockburn on a continuous visual tagged analogue scale using the 5 pictures provided in the Welfare Quality Protocol^®^ [26]. Specifically, a line of 100 mm was overlaid on the pictures where 0 referred to no lesions and 100 referred to maximum damage. A mark on this line was made using the pictures at positions 10 (original score 1) etc. During scoring the observer was unaware of the speed in the runway tests. Individual fecal samples were collected for analysis not reported here before the chick was returned to the catching frame. When all broilers had completed the runway tests, the water test was performed in four batches of 4 birds each.

### 2.4. Runway Tests

**Figure 1:**
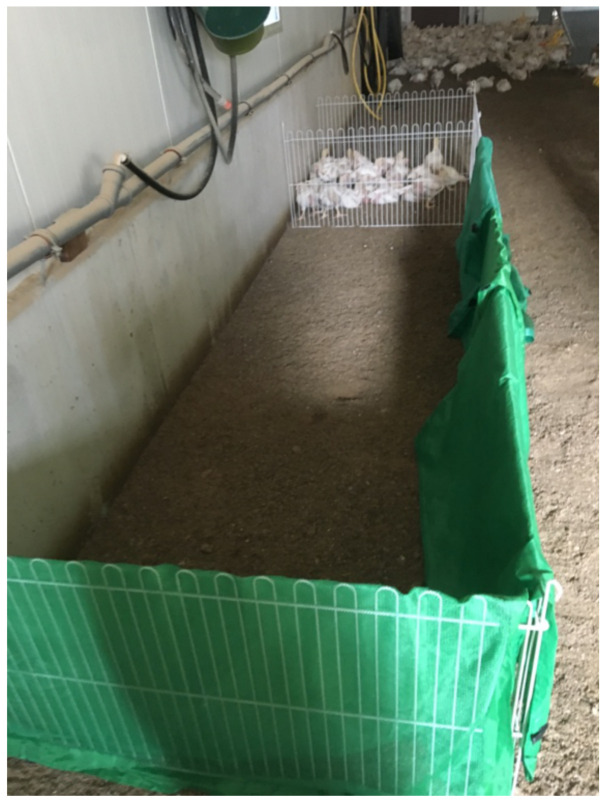
Runway where a chicken was taken from the holding pen (far end) and released at the near end of the runway. The time measured was from release until the chicken came within a bird’s length of its conspecifics in the holding pen, as assessed by the end of the green screen.

The runway used here consisted of a 342 cm long runway with opaque sides except the far end side where the chicks in the catching frame were visible (Figure 1). The opaque cover at the side ended one bird length before the catching frame and this was taken as the finish line. One chick at a time was carried to the end of the runway and then released. The time to reach the finish line was recorded with a timeout of 5 min. Immediately afterwards, the test was repeated by adding a line of bricks 14 cm high across the width of the runway about 50 cm from the release point and again the time to reach the finish line was recorded with a timeout of 5 min.

### 2.5. Water Test

The method was the “latency to lie” test [24,25]. Four birds were placed in a box covered with tepid shallow water and watched for 15 min. The time when a bird sat down for the first time was recorded. Broilers that did not sit down were scored with the maximum time of 900 s.

### 2.6. Optical Flow for Group Level Behaviour Assessment

Two Samsung CCTV IP cameras (SNO-6084RP) were fixed to the ceiling at a height of 5 m on both sides of the barn about one third of the total length of each house away from the entrance. Cameras were installed between flocks and were connected to a Synology NAS Disk Station (DS 115j) for video storage via an ethernet switch (HP 9982A). Videos were recorded 24 h/day from population until a few days before depopulation with 4 images/s, with a resolution of 320 × 240 pixels.

The movements of flocks were analyzed from the output of the cameras by detecting the rate of change of image brightness (“optical flow”) in different parts of the whole camera image both through time and space [27,28]. The resulting changes in different parts of an image were then combined to give an estimate of the sum of local velocity vectors. For example, if the entire flock of white chickens on a dark background remained stationary from one frame to the next, there would be no change of brightness and no “flow”. But if some chickens moved between frames, some of the white areas would become darker and vice versa and this would be registered as a net “flow”.

Optical flow can be detected down to the pixel level but for reasons of economy, each frame was divided into 1200 (40 × 30) 8-by-8 pixel blocks and the optical flow estimated for each block every 0.25 s. These estimated flow velocities were then combined, on a frame-by-frame basis to give the total “flow” over the entire image expressed as the mean optical flow (indicating overall average movement) plus variance, skew and kurtosis as different descriptors of variations of movement. Further details are given in [20].

To reduce the output to a manageable size, the data from 4 frames per second of one camera were aggregated into sequences of 3600 frames, giving average values of the four optical flow variables (mean, variance, skew and kurtosis) that represented 15 minutes of real time. Median values were used to eliminate spuriously large numbers that occasional occurred in the optical flow records due to artefacts. These 15 min summaries were then averaged to give the daily (08.00–20.00 h) values used for the comparison with behavior tests. To ensure that the comparison between different flocks was valid, it was important to take optical flow values for the same day of life for all flocks. Day 28 was chosen as this was the first day when behavior tests for all flocks had been completed and so optical flow values could be obtained with no disturbance from the behavior tests. Day 28 was before thinning for all flocks (see Section 2.2). 

### 2.7. Statistical Analysis

The first analysis used the measurements from individual birds to analyze the effects of body weight, sex, hockburn and pododermatitis on the time taken to complete the runway with and without obstacles. The times were logarithmically transformed and birds that did not complete the task (i.e., the measured times were above 300 s) were discarded. Hockburn and pododermatitis were analyzed as binary variables, being either 0 or more than 0. A generalized linear model (Proc Glimmix, SAS Institute Inc., Cary, NC, USA) was used for this analysis. Residuals were checked for normality. The full model included all interactions apart from those deleted when their *p*-level was above 0.2.

For logistical reasons, not all flocks could be tested at exactly the same age. As age and body mass in these rapidly growing birds are strongly confounded, we decided to include body mass instead of age in the models. Based on existing literature, e.g. [29], body mass was more likely to influence mobility than age per se (e.g., cognitive changes over a 5 day period).

The second analysis involved comparing the 5 measurements on individual birds (from the three behavior tests plus hockburn and pododermatitis scores) with the optical flow result from the flocks from which they were taken. To make the comparison between these two different kinds of data valid (16 individuals/flock), results of the 16 birds selected as a sample from each flock were first combined to give a single average value for that flock for each of the five measures. In contrast to the first analysis which used a binary score for hockburn and pododermatitis, this second analysis used the original continuous scores of hockburn and pododermatitis. To avoid making invalid assumptions about the distribution of data or equality of variances between the two datasets, a non-parametric test of correlation, Spearman Rank Correlation, was used [30].

## 3. Results

### 3.1. Statistical Description of Flock Movement

The optical flow description of the 18 Swiss flocks at 28 days of age is summarized in Table 1. All flocks exhibited a movement distribution with a strongly positive kurtosis, indicating a higher central peak and/or longer “tails” than would be expected in a normal distribution. Kurtosis is measured in standard deviations away from the mean and anything above (or below) 3 constitutes a departure from normality [15]. All flocks also exhibited a positive skew, showing that the mode was displaced to the left of the mean, with the “tail” of the distribution to the right.

### 3.2. Runway Tests

46/319 birds failed to reach the finish line within 5 min in the runway test without obstacles, and 79/319 failed in the runway test with obstacles and were recorded as having a time of 300 s. The time to finish the runway test with obstacles decreased with increasing body mass (F_1,282_ = 3.93, *p* = 0.049) and female chicks tended to take longer to finish the runway with obstacles (F_1,282_ = 3.47, *p* = 0.064). The same pattern applied to the runway without obstacles but these were non-significant trends (body mass: F_1,282_ = 3.63, *p* = 0.058; sex: F_1,282_ = 3.42, *p* = 0.066). Both pododermatitis and hockburn had *p*-values > 0.9 and were dropped from the final model for the runway tests with and without obstacles.

Body mass but not pododermatitis or hockburn significantly affected the time to complete the runway test, while the males tended to be faster than females (mass: F_1,236_ = 5.93, *p* = 0.016; sex: F_1,236_ = 3.06, *p* = 0.082, pododermatitis: F_1,236_ = 0.68, *p* = 0.41, hockburn: F_1,236_ = 2.79, *p* = 0.10). 

### 3.3. Latency to Lie (Water Test)

193/304 of birds remained standing at the end of the test and so were recorded as taking 900 s.

Heavier birds of both sexes sat down sooner in the water than lighter birds, whereas sex, pododermatitis, and hockburn were not associated with time to sit (body mass: F_1,275_ = 3.97, *p* = 0.047; sex: F_1,275_ = 0.11, *p* = 0.74; pododermatitis: F_1,275_ = 2.27, *p* = 0.13; hockburn: F_1,275_ = 0.08, *p* = 0.78).

### 3.4. Correlations between Individual Tests and Flock Optical Flow Statistics

The correlations between the three individual behavior tests and the day 28 optical flow values of the flocks from which the individual birds had been taken are shown in Table 2. In the two runway tests, there was a significant positive correlation between the mean time taken to move through the runway and both the skew and kurtosis of the flocks from which those individuals were taken. The birds that took the longest time in the runways came from the flocks with the highest positive skew and the highest kurtosis. In the water test, there was a significant positive correlation between the mean length of time standing and the mean and variance of optical flow. The birds that on average stayed standing the longest in the water test came from flocks with the highest mean rate of movement.

## 4. Discussion

The optical flow patterns produced by the movement of broiler chicken flocks showed a positive skew (Table 1), indicating that the mode of the flock movement distribution was displaced to the left and was lower than the mean. All flocks in this study also showed a highly positive kurtosis or right hand “tail” to the distribution, showing that at any one time, there was a small amount of movement that was much higher than the mean activity. Optical flow patterns therefore indicate that broiler chicken flocks investigated here consisted of a majority of birds that were relatively inactive for most of the time, with a small number of very active birds. This corresponds well with results of studies involving direct behavioral observations that show that individual broiler chickens may spend up to 90% of their time sitting or lying and that flocks are typically made up of a majority of inactive birds, with only the minority actively walking or running at any one time [31,32,33].

We tested the hypothesis that the more active birds, as assessed in individual behavior tests, would come from flocks with higher mean, but lower skew and lower kurtosis optical flow [6,17,18,19,20]. The two sorts of test we used in this study measured activity in opposite ways so it takes a moment’s thought to see that our hypothesis actually predicted correlations with opposite signs depending on whether we were using the runway test or the water test. In the runway test, activity was measured by how quickly a bird moved to the end of the runway: very active birds did this in a short time. We therefore predicted that there should be a negative correlation between time to complete the runway and mean optical flow in their home flock, but a positive correlation between time to complete the runway and skew and kurtosis of the optical flow of the flock. This prediction was partly fulfilled (Table 2). The average times the sample of 16 birds took to complete the runway tests were positively correlated with skew and kurtosis of flock movement, but were not significantly negatively correlated with mean optical flow (although they were in the predicted direction). A likely reason for this is that both the skew and kurtosis are particularly sensitive to the activity of the most active birds [15], whose activity was most accurately assessed in the runway tests, while the mean is more sensitive to the average movement of the least active birds, many of which (15.5% in Test 1 and 28.5% in Test 2) did not get to the end of the runway in the specified time and were simply measured as all having a time of above 300 s. This could have obscured any differences between the least active birds.

In the water test, on the other hand, our hypothesis made the predictions with the opposite sign. The water test measured the length of time birds are able to remain standing: the most active birds remained standing for the longest time. For this test, we predicted a positive correlation between the time spent standing and the mean level of activity in the flock from which birds were taken. This prediction was fulfilled (Table 2). There was a significant positive correlation between average time spent standing in the water test and the mean optical flow of the home flock. However, the predicted negative correlation between average time spent standing and both skew and kurtosis of flock optical flow, although in the predicted direction, this did not reach significance. A possible explanation for this is that the water test was much better at discriminating between the birds that were least able to stand but did not discriminate accurately between the most active birds because the majority (65.5%) remained standing at the end of the test and so their standing times all had to be scored as >900s. Taking both the two runway and the water tests together and allowing for each of the time limitations involved, our predicted correlations with flock optical flow measures were found. Where predicted correlations did not reach significance, this can be explained by the number of birds failing to complete the tests.

Negative welfare outcomes such as mortality, lameness and hockburn, are associated with reduced mean and higher skew and kurtosis of optical flow [6,15,18,19,20,21]. The behavior tests reported here confirm that low mean, high skew/high kurtosis flocks were the most likely to contain slow moving individuals that took a long average time to complete the runway and sat down after a short time standing in water. High kurtosis is associated with slow movement because in a flock of largely slow-moving birds, very active individuals stand out as an unusual minority and their movements appear as a long “tail” or high kurtosis in the movement distribution. The more birds that are active, the more normal active movement becomes and the lower the kurtosis that is recorded.

The lack of any detectable effect of pododermatitis or hockburn on the outcome of the behavior tests could be due to low power caused by a low sample size (only 16 chicks per flock), a low variation within flocks, or more relevant factors (e.g., variation in body mass) among flocks masking any influence of pododermatitis and hockburn. The behavior of the runway test was not only a measure of physical ability but also of motivation, which could have been influenced by other welfare parameters like anxiety. More anxious chicks might have been more likely to approach conspecifics quickly. We did not test that so we cannot draw any conclusions.

The average level of activity within a flock has been proposed as a general indicator of the welfare including health of a flock [34] and can easily be automatically detected as mean level of optical flow [6,7,10]. However, on its own, average flock activity can be difficult to interpret in welfare terms as it can be influenced by other factors such as breed and light levels. Crucially, it also fails to give any information about the welfare of different individuals within the flock [13]. Our results show that skew and kurtosis of optical flow provide additional information about the proportions of birds within a flock to which the average flock measures apply. A flock that is active (high mean) but where the most active birds show only slightly more movement than the rest of the flock (relatively low skew and kurtosis), is a flock where most individuals are active. A flock with a high positive skew and kurtosis on the other hand, indicates a flock where the level of movement of the majority of the flock is below average and a healthy active movement is shown of only a tiny minority. Our results that suggest skew and kurtosis of optical flow allow us to differentiate flocks that have different proportions of active and less active individuals.

## 5. Conclusions

Patterns of optical flow made by the movements of chicken flocks provide a group measure of flock behaviour in that they do not involve identifying or tracking individual birds. However, by individually testing a sample of birds from different flocks for their activity, we have shown that these group-level patterns also contain valuable information about the proportions of active and less active individuals within a flock and so provide a link between group and individual approaches to chicken behaviour.

## Figures and Tables

**Table 1:** Day 28 mean daily optical flow values for 18 flocks with the standard errors in brackets.

Mean	Variance	Skew	Kurtosis
0.24 (±0.24)	0.216 (±0.215)	4.762 (±4.77)	30.109 (±3.25)

**Table 2:** Spearman correlations between behavior tests and optical flow (OF).

	Mean OF	Variance OF	Skew OF	Kurtosis OF
Runway test 1 (without obstacles)	−0.0781	0.008	0.711 *p* < 0.001	0.608 *p* < 0.01
Runway test 2 (with obstacles)	−0.196	−0.05	0.697 *p* < 0.0025	0.625 *p* < 0.005
Water test	0.573 *p* < 0.01	0.574 *p* < 0.01	−0.308	−0.343
Hockburn	−0.021	0.147	0.508 *p* < 0.025	0.508 *p* < 0.025
Pododermatitis	0.023	−0.003	0.398	0.426 *p* < 0.05

## Data Availability

Data collected on farms belongs to the farmers concerned and was made available to us only with their permission. An anonymized version of the data will be made available on application to the authors.
